# The impact of 3-sulfo-taurolithocholic acid on ATPase activity in patients’ colorectal cancer and normal colon tissues, and its hepatic effects in rodents

**DOI:** 10.3389/fvets.2024.1480122

**Published:** 2024-12-05

**Authors:** Solomiia Bychkova, Mykola Bychkov, Dani Dordević, Simon K.-M. R. Rittmann, Monika Vítězová, Ivan Kushkevych

**Affiliations:** ^1^Department of Human and Animal Physiology, Faculty of Biology, Ivan Franko National University of Lviv, Lviv, Ukraine; ^2^Department of Therapy No. 1, Medical Diagnostic and Hematology and Transfusiology of Faculty of Postgraduate Education, Danylo Halytsky Lviv National Medical University, Lviv, Ukraine; ^3^Department of Plant Origin Food Sciences, Faculty of Veterinary Hygiene and Ecology, University of Veterinary Sciences Brno, Brno, Czechia; ^4^Archaea Physiology & Biotechnology Group, Department of Functional and Evolutionary Ecology, Universität Wien, Wien, Austria; ^5^Department of Experimental Biology, Faculty of Science, Masaryk University, Brno, Czechia

**Keywords:** colorectal cancer, colon mucosae, Na^+^/K^+^ ATPase, Ca^2+^ ATPase, basal Mg^2+^ ATPase, bile acid

## Abstract

Colorectal cancer is influenced by genetic mutations, lifestyle factors, and diet, particularly high fat intake, which raises bile acid levels in the intestinal lumen. This study hypothesized that bile acids contribute to tumorigenesis by disrupting ion transport and ATPase activity in the intestinal mucosa. The effects of 3-sulfo-taurolithocholic acid (TLC–S) on ATPase activity were investigated in colorectal cancer samples from 10 patients, using adjacent healthy tissue as controls, and in rodent liver function. ATPase activity was measured spectrophotometrically by determining inorganic phosphorus (P_i_) in postmitochondrial fractions. Ca^2+^ dynamics were assessed in isolated mouse hepatocytes with fluorescence imaging, and rat liver mitochondria were studied using polarographic methods to evaluate respiration and oxidative phosphorylation. TLC–S increased Na^+^/K^+^ ATPase activity by 1.5 times in colorectal cancer samples compared to controls (*p* ≤ 0.05). In healthy mucosa, TLC–S decreased Mg^2+^ ATPase activity by 3.6 times (*p* ≤ 0.05), while Mg^2+^ ATPase activity in cancer tissue remained unchanged. TLC–S had no significant effect on Ca^2+^ ATPase activity in healthy colon mucosa but showed a trend toward decreased activity in cancer tissue. In rat liver, TLC–S decreased Ca^2+^ ATPase and Na^+^/K^+^ ATPase activities while increasing basal Mg^2+^ ATPase activity (*p* ≤ 0.05). Additionally, TLC–S induced cytosolic Ca^2+^ signals in mouse hepatocytes, partially attenuated by NED-19, an NAADP antagonist (*p* ≤ 0.05). TLC–S also reduced the V3 respiration rate of isolated rat liver mitochondria during *α*-ketoglutarate oxidation. These findings suggest that TLC–S modulates ATPase activity differently in cancerous and healthy colon tissues, playing a role in colorectal cancer development. In rat liver, TLC–S affects mitochondrial activity and ATPase function, contributing to altered cytosolic calcium levels, providing insight into the mechanistic effects of bile acids on colorectal cancer and liver function.

## Introduction

Bile acids (BAs) are synthesized from cholesterol by hepatocytes in the liver and are essential components of bile, which aids in the emulsification and absorption of fats when excreted into the duodenum. Elevated fat consumption has been linked to increased levels of BAs in the intestinal lumen, where they promote the growth and activity of bacteria that convert primary BAs into secondary bile acids (SBAs) with potent tumorigenic properties (1–3). SBAs, such as deoxycholic acid and lithocholic acid, are implicated in the development of colon cancer (4). These compounds are formed in the intestine through bacterial enzymatic actions on primary bile acids secreted by the liver, namely chenodeoxycholic acid and cholic acid (5). Deoxycholic acid and lithocholic acid, in particular, possess potentially carcinogenic properties and can induce DNA damage in intestinal epithelial cells through the generation of reactive oxygen species (ROS), leading to mutations and cancer cell proliferation (4, 6). Moreover, SBAs can modulate cellular signaling pathways such as the Wnt/*β*-catenin pathway and NF-κB, promoting cell proliferation, inhibiting apoptosis, and inducing inflammation, all of which contribute to carcinogenesis (7). SBAs also stimulate the production of pro-inflammatory cytokines, leading to chronic intestinal inflammation, a known risk factor for colorectal cancer (8). Alterations in the gut microbiota composition under the influence of SBAs may further exacerbate cancer development by affecting immune and inflammatory responses (9).

Given the pivotal role of SBAs in colon cancer pathogenesis, further research is warranted to explore preventive and therapeutic strategies (7). BAs also play a crucial role in regulating ATPases’ activity in the intestinal mucosa, particularly Na^+^/K^+^ ATPase and Ca^2+^ ATPase. Na^+^/K^+^ ATPase is essential for maintaining electrolyte balance and cell membrane potential and has been implicated in cancer cell adhesion, motility, and migration (10, 11). It has been demonstrated that Na^+^/K^+^ ATPase facilitates BAs absorption by cells in the ileum through the maintenance of the Na^+^ electrochemical potential gradient for coupled bile acid transport (12). Inhibition of Na^+^/K^+^ ATPase induces hybrid cell death and enhanced sensitivity to chemotherapy in human glioblastoma cells (13). However, its role in cancer progression under BAs remains poorly understood.

Ca^2+^ ATPases are responsible for establishing Ca^2+^ gradients across cellular membranes and maintaining low cytoplasmic Ca^2+^ levels, with alterations in Ca^2+^ signaling being implicated in cancer progression (14–16). Changes in the expression of plasma membrane Ca^2+^ pumps (PMCA) and associated calcium modifications have been observed in colon cancer, facilitating cancer cell proliferation (14). Colorectal cancer cell differentiation has been linked to changes in Ca^2+^ homeostasis and the expression of specific isoforms of the endoplasmic reticulum (ER) Ca^2+^ ATPase (14). Dysregulation of sarco/endoplasmic reticulum Ca^2+^ ATPase (SERCA) expression and activity can induce ER stress and apoptosis, contributing to cancer progression (17). Basal Mg^2+^ ATPase represents another system of active ion transport in cells, influencing proton content in extracellular or intracellular spaces.

This study aimed to investigate the impact of 3-sulfo-taurolithocholic acid (TLC–S) on ATPase activity in patients’ colorectal cancer samples, adjacent healthy colon mucosae serving (controls) and its effects on rat liver. Additionally, we examined the effects of TLC–S on mitochondrial respiration of isolated rat liver mitochondria and on cytosolic and stored Ca^2+^ in isolated mouse hepatocytes.

## Materials and methods

### Standards of ethics and patient attributes

All animal procedures followed the guidelines outlined in the “International Convention for Animal Research” and were approved by the Bioethics Committee of the Biological Faculty at the Ivan Franko University of Lviv, under Protocol No. 02/03, 2024.

Human sample collection adhered to the principles of the Declaration of Helsinki and was approved by the Institutional Review Board (Ethics Committee) of the Department of Therapy No. 1, Medical Diagnostics, Hematology, and Transfusion Medicine at the Faculty of Postgraduate Education, Danylo Halytsky Lviv National Medical University (Protocol Code No. 2, March 3, 2024). A total of 10 patients diagnosed with colorectal cancer (average age 54.3 ± 1.7 years) participated in the study. Informed consent was obtained from all patients before obtaining colonic mucosa samples during endoscopy. Samples were collected from both cancer-affected areas and healthy areas (control) of the colonic mucosa from each patient.

### Postmitochondrial subcellular fraction preparation

Postmitochondrial subcellular fraction was obtained as described early (18–20). Briefly, to obtain the postmitochondrial subcellular fraction, tissue samples (including colon mucosae from patients, colorectal cancer samples, and rat liver) were homogenized in a Potter–Elvehjem homogenizer at 300 rpm using a buffer solution at a 1: 8 mass ratio. The postmitochondrial subcellular fraction was isolated via differential centrifugation, which involves sequential centrifugation steps to separate organelles and membrane fragments from the homogenized tissue. Initially, the homogenate was centrifuged at 3000 g for 10 min (using an RS-6 centrifuge) to pellet intact cells and nuclei. Subsequently, the mitochondrial fraction was pelleted by centrifuging at 6,500 g for 10 min at a temperature of 0–2°C. The resultant postmitochondrial supernatant was then used as the subcellular fraction for further experiments.

### Measurement of ATPase activity

At the beginning of the experiment, 200 μL of the subcellular fraction of the tissue (*patients colon mucosae or colorectal cancer samples or rat liver*) was placed in an incubation medium without ATP, which contained (mmol/L): NaCI – 50.0; KCI – 100.0; tris/NSI – 20.0; MgCl_2_–3.0; CaCl_2_–0.01; NaN_3_–1 (selective inhibitor of mitochondrial ATPase); pH = 7.4 at 37°C. The ATP hydrolase reaction was initiated by adding 3 mmol/L ATP (Sigma) and the samples were incubated for 15 min at 37°C with moderate shaking in a water ultra-thermostat. TLC–S was added to postmitochondrial subcellular fraction at a concentration 50 μmol/L. After completion of the incubation, the reaction was stopped by adding 5 mL of 10% trichloroacetic acid, the samples were kept for 10 min and centrifuged at 1,600 g for 10 min. The resulting protein–free supernatant was used to determine the content of inorganic phosphorus (P_i_) in it by the Fiske–Subarrow method. The ATPase activity of the subcellular fraction of the liver was calculated by the difference in the content of P_i_ in media of different composition and expressed in μg P_i_/hour × mg^−1^ of protein (21). The total ATPase activity of the subcellular fraction was determined in Ca^2+^– and Mg^2+^–containing incubation medium. To determine the specific Na^+^/K^+^ ATPase activity, the ATPase activity of the subcellular fraction was subtracted from the total ATPase activity in a medium containing 1 mmol/L ouabain (Sigma). The specific Mg^2+^ ATPase activity was determined in the incubation medium containing 1 mmoL/L EGTA in the absence of CaCl_2_ and the presence of 1 μmol/L ouabain. Thapsigargin (Sigma) at a concentration of 1 μmol/L was used to isolate the contribution of the Ca^2+^ pump to the EPR. In all experiments, the control for non-enzymatic ATP hydrolysis was a reaction medium in which no subcellular fraction was added.

### Isolation of rat liver mitochondria and measurement of respiration rate in isolated rat mitochondria

The method was described before (22). Experiments were conducted on non-linear male rats weighing 180–200 g. The rats were humanely euthanized. The liver was excised and briefly perfused with a homogenization medium containing 250 mmol/L sucrose, 1 mmol/L EDTA, and 10 mmol/L Tris/NSI (pH 7.4, 4°C). The cooled liver tissue was then minced using a press.

The liver homogenate was centrifuged at 3,000 g for 10 min using a Jouan Mr. 1812 centrifuge (Jouan, France) to pellet nuclei, large cell fragments, and intact cells, while mitochondria remained in the supernatant. The isolated mitochondria were incubated in a polarographic chamber containing a medium with 250 mmol/L sucrose, 10 mmol/L HEPES, 1 mmol/L EGTA, 1 mmol/L KH_2_PO_4_, and 1 mmol/L MgCl_2_, at pH 7.2. *α*-ketoglutarate (1 mmol/L) and succinate (0.35 mmol/L) were used as exogenous oxidative substrates. TLC–S was added at the same concentration as described previously. The rate of oxygen consumption and the efficiency of ATP synthesis in various metabolic states were assessed using a polarographic technique with a Clark electrode (23–25). In the “resting” state (State 2), an exogenous substrate was added to the mitochondria, resulting in a slow respiration rate (V2), zero phosphorylation rate, and a significantly reduced electron flow in the respiratory chain. In the “active” state (State 3), mitochondrial respiration (V3) was sharply stimulated by the addition of 50 μmol/L ADP, accompanied by ATP synthesis and an increase in the concentration of oxidized respiratory intermediates. In the “controlled” state (State 4), the complete conversion of ADP to ATP occurred in the polarographic chamber, with a high substrate concentration maintained, resulting in a decreased oxygen consumption rate (V4) compared to State 3.

### Isolation of mouse hepatocytes

Male CD-1 mice were humanely euthanized. The isolated liver was perfused with buffer without Ca^2+^: 140 mmol/L NaCl; 4.7 mmol/L KCl; 10 mmol/L HEPES; 10 mmol/L D-glucose; 100 μmol/L EGTA; pH 7.4; the perfusion rate was 5 mL/min at 37°C. The liver was then perfused with buffer in the presence of 1.3 mmol/L CaCl_2_ and collagenase I (Worthington) for 10 min at 37°C. Dissociated hepatocytes were centrifuged at 50 × g for 1 min and then transferred to a buffer containing 1 mmol/L MgCl_2_ and 1.3 mmol/L CaCl_2_, pH 7.4.

### Fluorescence measurement of [Ca^2+^] in the cytosol of mouse hepatocytes

After isolation, mouse hepatocytes were loaded with the low-affinity Ca^2+^ sensitive dye fluo-4 (2.5 μmol/L) for 30–45 min at 36.5°C. The cells were then attached to poly-L-lysine-coated coverslips in a flow chamber, and all experiments were conducted at room temperature.

Fluorescence images were acquired using a Leica SP2 MP dual two-photon confocal microscope with a 63x/1.2 NA objective. For fluo-4, excitation was performed at 488 nm (argon ion laser, 3% power) and emission was collected between 510–590 nm. Fluorescent images were recorded at a frequency of 0.6–1.0 frames per second. Fluorescence signals were expressed as F/F_0_, where F represents the fluorescence intensity during the experiment and F_0_ is the initial fluorescence intensity.

### Measurement of [Ca^2+^] in intracellular stores of permeabilized mouse hepatocytes

A suspension of permeabilized mouse hepatocytes (2 × 10^6^ cells) was loaded with the fluorescent dye Mag-Fura-2 AM (5 μmol/L). The dye was removed before permeabilization. Hepatocytes were permeabilized with 0.1 mg/mL saponin in an intracellular solution for 10 min. Subsequently, the cells were washed with an intracellular solution based on K–HEPES, containing 20 mmol/L NaCl, 127 mmol/L KCl, 1.13 mmol/L MgCl_2_, 0.05 mmol/L CaCl_2_, 0.1 mmol/L EGTA, 10 mmol/L HEPES (KOH), 5 μg/mL oligomycin, 1 μg/mL rotenone, and 2.0 mM ATP, at pH 7.0. A 2 mL aliquot of the cell suspension was transferred to the spectrofluorometer cuvette. Mag-Fura-2 AM fluorescence was monitored with excitation at 340–380 nm and emission at 500 nm. The intracellular calcium content mobilized by 10 μmol/L ionomycin was considered as 100% and represented the total Ca^2+^ in the internal pool.

### Statistical analysis

The data presented in the text are expressed as mean ± SEM. An ANOVA test was conducted to determine the significance of differences between experimental groups, with *p* < 0.05 considered significant. The analysis was performed using the statistical software Origin Pro 2018.

## Results

### ATPase activity of patient’s colon mucosae and colorectal cancer tissue samples under TLC–S adding

**Figure 1:**
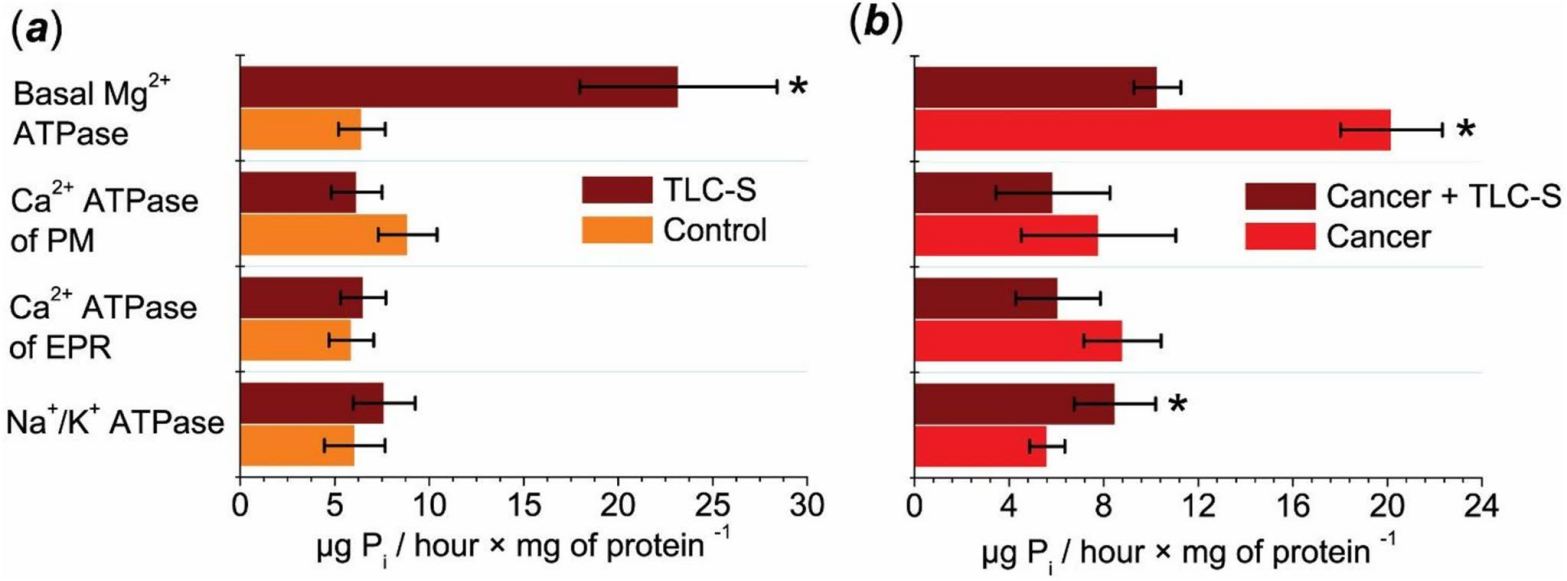
Effect of TLC–S on ATPase activity in the subcellular postmitochondrial fraction of patients’ mucosae tissue (M ± m): (a) control refers to mucosae tissue samples without TLC–S treatment (*p ≤ 0.05 vs. control) and (b) patients’ cancer tissue samples: “cancer” refers to adenocarcinoma of patients’ colon tissue samples without TLC–S treatment (*p ≤ 0.05 vs. cancer).

During the action of TLC–S on the control (normal tissue of colon mucosae), the activity of the Na^+^/K^+^ pump did not undergo statistically significant changes (Figure 1a). In colorectal cancer samples, the specific activity of Na^+^/K^+^ ATPase under the effects of TLC–S increased from 5.61 ± 0.74 to 8.48 ± 1.72 μg P_i_/hour × mg^−1^ of protein. Therefore, TLC–S causes a statistically significant 1.5-fold increase in the activity of Na^+^/K^+^ ATPase in the subcellular fraction of colorectal cancer samples (Figure 1b). In normal tissue, the Na^+^/K^+^ ATPase activity under the influence of TLC–S was 7.62 ± 1.64 μg P_i_/hour × mg^−1^ of protein, which is slightly lower than in the cancer samples (8.48 ± 1.72 μg P_i_/hour × mg^−1^). Thus, the increase in Na^+^/K^+^ ATPase activity was more pronounced in the cancer tissue compared to the normal tissue after TLC–S treatment.

The addition of TLC–S to the incubation medium of normal tissue samples caused a slight increase in the specific activity of ER Ca^2+^ ATPase, which was not statistically significant (Figure 1a). In cancer tissue samples, the ER Ca^2+^ ATPase activity decreased from 8.8 ± 1.63 μg P_i_/hour × mg^−1^ of protein to 6.07 ± 1.79 μg P_i_/hour × mg^−1^ of protein upon the addition of TLC–S. Therefore, the action of TLC–S resulted in a consistent trend of decreased EPR Ca^2+^ ATPase activity in the subcellular fraction of human colorectal cancer samples (Figure 1b). In normal tissue, the ER Ca^2+^ ATPase activity after TLC–S treatment was 6.51 ± 1.20 μg P_i_/hour × mg^−1^ of protein, while in cancer tissue, it decreased to 6.07 ± 1.79 μg P_i_/hour × mg^−1^. This suggests a slightly more prominent reduction in cancer tissues compared to normal ones following the addition of TLC–S.

It was found that with the addition of TLC–S to the incubation medium of the control samples, the activity of the PM Ca^2+^ pump did not change statistically authentic (Figure 1a). Thus, we observed only a trend toward a decrease in the activity of the PM Ca^2+^ ATPase in the subcellular fraction of normal cells under the influence of TLC–S. In colorectal cancer tissue samples, the specific activity of the PM Ca^2+^ ATPase was 7.79 ± 3.26 μg P_i_/hour × mg^−1^ of protein. The addition of TLC–S resulted in a decrease in the activity of this pump to 5.85 ± 2.41 μg P_i_/hour × mg^−1^ of protein (Figure 1b).

In normal tissue, PM Ca^2+^ ATPase activity under TLC–S was 6.16 ± 1.34 μg P_i_/hour × mg^−1^ of protein, while in cancer tissues it was 5.85 ± 2.41 μg P_i_/hour × mg^−1^. This shows a comparable trend toward decreased activity in both tissue types, although the reduction was slightly more pronounced in cancer tissues.

In the control samples, we observed a statistically significant 3.6-fold increase in the activity of basal Mg^2+^ ATPase in the subcellular fraction of normal tissue under the influence of TLC–S compared to the samples with lack of TLC–S (Figure 1a). In colorectal cancer tissue, the activity of basal Mg^2+^ ATPase under the influence of TLC–S ranged from 5.16 to 13.06, averaging 10.27 ± 0.99 μg P_i_/hour × mg^−1^ of protein. In the absence of TLC–S in the incubation medium, the activity of basal Mg^2+^ ATPase ranged from 3.78 to 25.63, averaging 20.17 ± 2.15 μg P_i_/hour × mg^−1^ of protein.

For basal Mg^2+^ ATPase, normal tissue showed a dramatic increase in activity to 23.19 ± 5.22 μg P_i_/hour × mg^−1^ of protein after TLC–S treatment, while in cancer tissue, the activity decreased to 10.27 ± 0.99 μg P_i_/hour × mg^−1^. This indicates a stark contrast between the tissue types, with normal tissue showing a substantial increase in activity, and cancer tissue showing a nearly twofold decrease. Thus, we found that TLC–S (50 μmol/L) causes a 1.96-fold decrease in the activity of basal Mg^2+^ ATPase in the subcellular fraction of human colorectal cancer samples (Figure 1b).

### Measurement of ATPase activity in the subcellular post-mitochondrial fraction of rat liver

**Figure 2:**
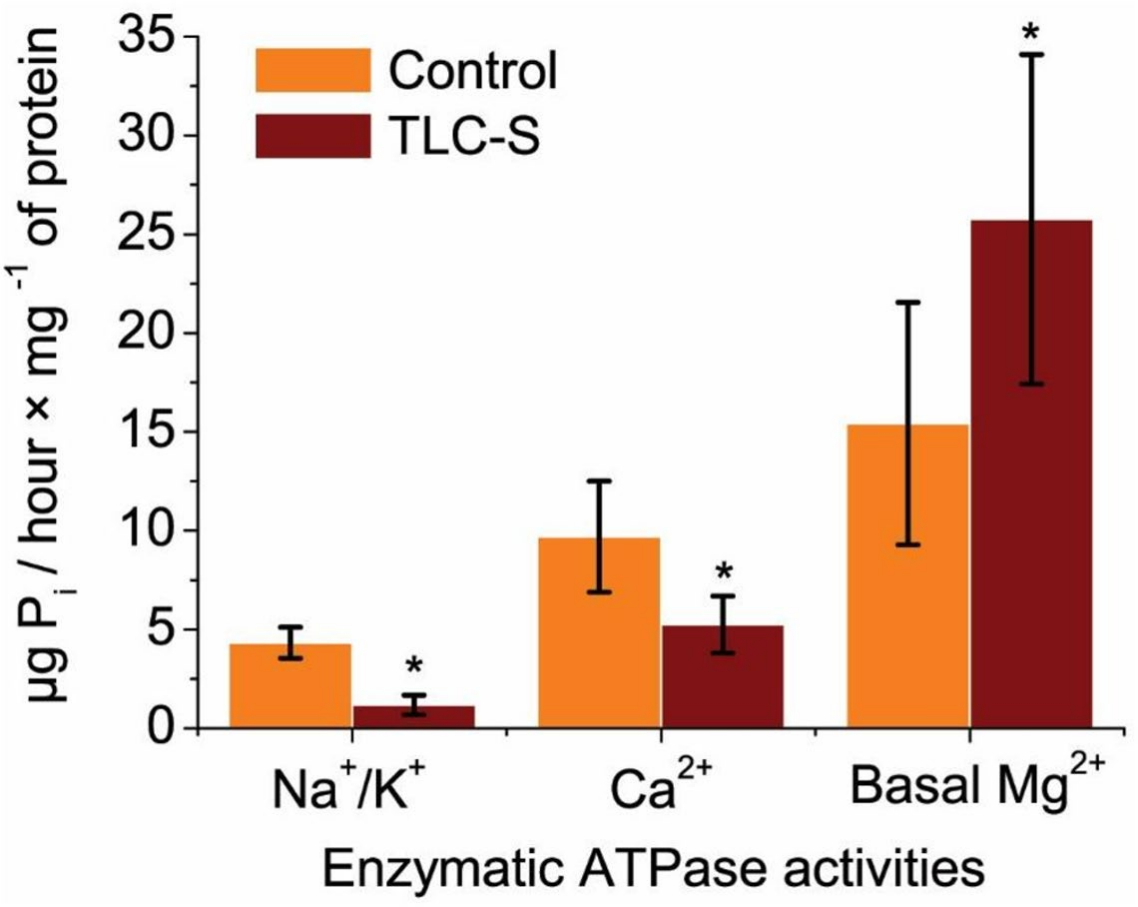
Effect of TLC–S on ATPase activity in subcellular postmitochondrial fraction on rat liver: each first column in the pair represents control without TLC–S (M ± m): *p ≤ 0.05 vs. control. Regarding Ca^2+^ ATPase, its activity in the control samples was measured at 9.70 ± 2.81 μg P_i_/hour × mg^−1^ of protein, whereas in samples incubated with TLC–S, it averaged around (5.25 ± 1.44) μg P_i_/hour × mg^−1^ of protein. We observed that TLC–S (50 μmol/L) reduced Ca^2+^ ATPase activity by 1.84 times (*p* ≤ 0.05; *n* = 5) in the subcellular fraction of rat liver, indicating a decrease in total Ca^2+^ ATPase activity in rat liver. Additionally, the basal activity of Mg^2+^ ATPase in control samples was approximately (15.42 ± 6.14) μg P_i_/hour × mg^−1^ of protein, while in samples incubated with TLC–S, it rose to (25.76 ± 8.34) μg P_i_/hour × mg^−1^ of protein. TLC–S (50 μmol/L) was found to increase basal Mg^2+^ ATPase activity by 1.67-fold (*p* ≤ 0.0.05; *n* = 5).

In the control group, the specific activity of Na^+^/K^+^ ATPase averaged approximately (4.32 ± 0.79) μg P_i_/hour × mg^−1^ of protein. Upon the addition of TLC–S (50 μmol/L) to the incubation medium, it decreased to (1.18 ± 0.50) μg P_i_/hour × mg^−1^ of protein. Thus, our findings indicate that TLC–S reduces the activity of Na^+^/K^+^ ATPase by 3.67 times (*p* ≤ 0.05; *n* = 5) (Figure 2).

### TLC–S–triggered elevation in cytosolic Ca^2+^ levels in intact hepatocytes preloaded with fluo-4

**Figure 3:**
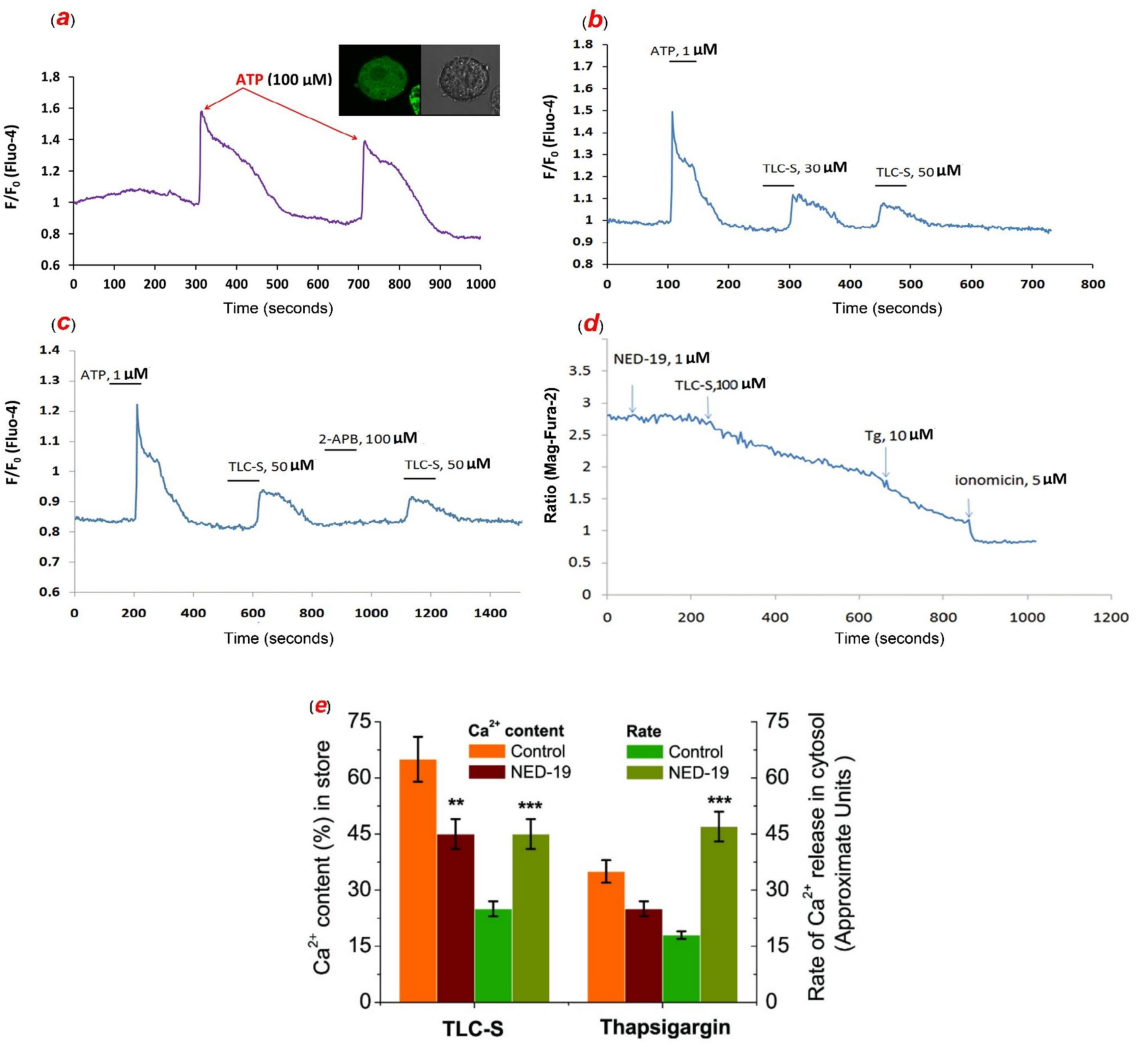
The effect of ATP on the content of Ca^2+^ in the cytosol of intact mouse hepatocytes loaded with fluo-4 (a–c) and stored Ca^2+^ inside of permeabilized mouse hepatocytes loaded with mag-fura-2 (d): a representative recording and photo of isolated hepatocytes loaded with the dye (a); TLC–S induced increase in cytosolic Ca^2+^ content in intact mouse hepatocytes loaded with fluo-4 and TLC–S induced increase in cytosolic Ca^2+^ content is concentration-dependent (30–50 μmol/L) (b); TLC–S induced increase in cytosolic Ca^2+^ content in intact mouse hepatocytes loaded with fluo-4 in the presence of 2-APB and 2-APB does not inhibit the TLC–S induced rise in cytosolic Ca^2+^ content (c); the effect of TLC–S on the content of sequestered Ca^2+^ in permeabilized mouse hepatocytes loaded with mag-fura-2 in presence of NED-19 (1 μmol/L) and TLC–S at a concentration of 100 μmol/L induces Ca^2+^ release from stores, subsequent application of thapsigargin causes depletion of the stores, and further use of ionomycin releases the remaining Ca^2+^ (d); the summarized data on the effects of TLC–S and thapsigargin on calcium storage, along with the average rate of calcium release triggered by TLC–S and thapsigargin, in the cytosol of mouse hepatocytes (e) (*** p ≤ 0.01 vs. control).

We demonstrated that TLC–S at concentrations of 50, 100, and 200 μmol/L elicited cytosolic Ca^2+^ responses, consistent with findings reported in prior studies (26, 27). A representative recording the effect of ATP on increasing the content of Ca^2+^ in the cytosol of intact hepatocytes loaded with fluo-4 and a photo of isolated hepatocytes loaded with the dye is shown in Figure 3a. Following the Ca^2+^ signal triggered by TLC–S, hepatocytes remained responsive to ATP, albeit with smaller amplitude and a longer plateau period (Figure 3b). It depicts a typical recording of repeated application of various concentrations of TLC–S. TLC–S induced increase in [Ca^2+^]_i_ is concentration-dependent (30–50 μmol/L). As can be observed in Figure 3c, 2-APB does not inhibit the TLC–S induced rise in [Ca^2+^]_i_ (Figure 3c).

### Effect of TLC–S on the content of stored Ca^2+^ in permeabilized mouse hepatocytes loaded with Mag-Fura-2

A representative recording depicting the impact of TLC–S on stored calcium levels using Mag-Fura-2 (5 μmol/L) (F/F_0_) was presented in Figure 3d. In suspensions of mouse permeabilized hepatocytes, TLC–S at a concentration of 100 μmol/L released approximately 66.10 ± 8.87% of the total accumulated calcium, which was comparable to the release induced by ionomycin (10 μmol/L). Following exposure to TLC–S, thapsigargin was able to release an additional 47.94 ± 13.05% of the total stored calcium. Upon application of NED-19 (1 μmol/L), the fraction of calcium released by TLC–S decreased to approximately 33.25 ± 2.15% of the total calcium released by ionomycin. Subsequent application of thapsigargin then mobilized only 21.75 ± 10.68% of the stored calcium in the suspension of mouse hepatocytes (Figure 3e). This suggests that pretreatment with NED-19 (1 μmol/L) likely reduced the calcium release induced by TLC–S by approximately 2-fold (*n* = 6; *p* ≤ 0.01). At the same time, we found that the rate of TLC–induced calcium release from the store increased 1.8-fold under the influence of NED-19, while rate of thapsigargin-induced calcium release increased 2.6-fold (*n* = 6; *p* ≤ 0.01) (Figure 3e).

### The effect of taurolithocholic acid on the respiration parameters of rat liver mitochondria

Following the addition of TLC to rat liver mitochondria for *α*-ketoglutarate oxidation, the mean values of respiratory states V_2_ decreased from 0.10 to 0.09 ng O/mg of protein, V_3_ decreased from 0.17 to 0.15 ng O/mg of protein, and V_4_ from 0.09 to 0.08 ng O/mg of protein. Comparing the respiratory rate indices between the control and TLC effects a significant 12% decrease in V_3_ state indicators was observed (*p* ≤ 0.05).

During respiratory parameter measurements for succinate oxidation with TLС–S addition, the mean respiratory state parameters V_2_ were 0.10 ng O/mg of protein, V_3_ averaged 0.22 ng O/mg of protein, and V_4_ averaged 00.06 ng O/mg of protein. Comparison of respiratory rates between control and TLС–S effects (Table 1) did not reveal statistically significant changes in respiratory rates due to succinate oxidation.

**Table 1:** Indicators of the effect of TLC–S on the respiratory rates (V_2_ V_3_ V_4_) of isolated rat liver mitochondria (M ± m).

	α-Ketoglutarate			Succinate		
	V_2_	V_3_	V_4_	V_2_	V_3_	V_4_
Control	0.10 ± 0.01	0.17 ± 0.01	0.09 ± 0.03	0.16 ± 0.04	0.34 ± 0.16	0.08 ± 0.02
TLC–S	0.09 ± 0.01	0.15 ± 0.01*	0.08 ± 0.01	0.10 ± 0.01	0.22 ± 0.04	0.06 ± 0.01

V_2_ corresponds to the “resting” state (State 2) of mitochondria, where the addition of an exogenous substrate leads to a slow respiration rate. V_3_ represents the “active” state (State 3) of mitochondrial respiration, induced by the addition of 50 μM ADP. V_4_ denotes the “controlled” state (State 4) of the mitochondria, wherein the complete conversion of ADP to ATP occurs within the polarographic chamber. *p ≤ 0.05 vs control.

Thus, in studying the impact of TLC–S on rat liver mitochondria respiration parameters, a significant decrease in V_3_ respiration rate during *α*-ketoglutarate oxidation was observed.

In our analysis, we also assessed several other key parameters of mitochondrial function, including respiratory control ratios (V_3_/V_2_ and V_3_/V_4_), ADP/O ratios, oxidative phosphorylation rates (V_f_), and phosphorylation time (T_f_). Respiratory control (Table 2), an indicator of mitochondrial integrity and coupling efficiency, was evaluated using two approaches: the Lardy method (V_3_/V_2_), which reflects the rate of oxygen uptake during ATP synthesis (V_3_) compared to the resting state (V_2_), and the Chance method (V_3_/V_4_), which compares respiration rates before and after ATP loading. The respiratory control values for both substrates (*α*-ketoglutarate and succinate) in the control group are above 1, indicating that the mitochondria are intact and functionally active. This confirms that the mitochondrial preparations used in the study were of good quality and had intact membranes.

**Table 2:** The impact of TLC–S on mitochondrial respiratory control ratios (Lardy method V_3_/V_2_ and Chance method V_3_/V_4_) for α-ketoglutarate and succinate oxidation of isolated rat liver mitochondria (M ± m).

Substrate	Control (V_3_/V_2_)	TLC–S (V_3_/V_2_)	Control (V_3_/V_4_)	TLC–S (V_3_/V_4_)
α-Ketoglutarate	1.83 ± 0.31	1.61 ± 0.06	2.02 ± 0.33	1.90 ± 0.21
Succinate	2.28 ± 0.23	1.79 ± 0.29	4.73 ± 0.55	3.11 ± 1.10

In our experiments (Table 2), TLC–S slightly reduced the V_3_/V_2_ ratio for α-ketoglutarate from 1.83 ± 0.31 to 1.61 ± 0.06, and for succinate from 2.28 ± 0.23 to 1.79 ± 0.29, but these changes were not statistically significant. Similarly, the V_3_/V_4_ ratio decreased from 2.02 ± 0.33 to 1.90 ± 0.21 for *α*-ketoglutarate and from 4.73 ± 0.55 to 3.11 ± 1.10 for succinate. This indicates that TLC–S might disrupts the coupling of respiration and oxidative phosphorylation during succinate oxidation, which could be a sign of initial mitochondrial membrane damage or a reduction in the efficiency of the electron transport chain. Thus, TLC–S affects mitochondrial function, reducing its efficiency, with a stronger impact observed during succinate oxidation compared to α-ketoglutarate.

The ADP/O ratios (Table 3), representing the efficiency of oxidative phosphorylation, remained stable or slightly increased (from 0.65 ± 0.02 to 0.84 ± 0.20 for α-ketoglutarate and from 0.67 ± 0.05 to 0.91 ± 0.10 for succinate). This suggests that TLC–S enhances the efficiency of oxidative phosphorylation, meaning that more ATP molecules are synthesized per oxygen molecule consumed. However, given the large standard deviation for the TLC–S group, this increase may not be statistically significant.

**Table 3:** The impact of TLC–S on rat liver mitochondrial respiratory parameters (ADP/O, Vf, and Tf) for *α*-ketoglutarate and succinate oxidation (M ± m).

Substrate	ADP/O (Control)	ADP/O (TLC–S)	Vf (Control)	Vf (TLC–S)	Tf (Control)	Tf (TLC–S)
α-Ketoglutarate	0.65 ± 0.02	0.84 ± 0.20	0.11 ± 0.01	0.12 ± 0.03	1874.19 ± 82.68	1724.51 ± 372.29
Succinate	0.67 ± 0.05	0.91 ± 0.10	0.23 ± 0.04	0.17 ± 0.07	1022.85 ± 288.54	1381.21 ± 565.75

The Vf value (Table 3) remains nearly unchanged under the influence of TLC–S (from 0.11 to 0.12), suggesting that TLC–S has little effect on the overall phosphorylation rate for α-ketoglutarate. For succinate, the Vf decreases from 0.23 to 0.17 with TLC–S treatment, indicating a reduction in the phosphorylation rate. Despite this reduction, the large standard deviation for TLC–S means the decrease might not be statistically significant without further analysis.

The phosphorylation time (Tf) slightly decreases from 1874.19 to 1724.51 under TLC–S, suggesting that mitochondria complete the phosphorylation cycle faster with α-ketoglutarate as the substrate (Table 3). However, the decrease is relatively small, and given the standard deviations, it might not be statistically significant. For succinate, Tf increases from 1022.85 to 1381.21 with TLC–S treatment, indicating a delay in the phosphorylation process. Given the large variation in the TLC–S group, this increase may not be statistically significant either.

## Discussion

Colorectal cancer (CRC) is the third most common cancer worldwide, with its etiology heavily influenced by nutrition, particularly a high-fat/high-carbohydrate Western diet. Other possible factor causing CRC development can be microbial composition and microbiome activity in the gut (20, 28–36). Epidemiological studies in both humans and animals have demonstrated that the risk of colon cancer is associated with BAs concentrations in feces. Elevated levels of BAs cause various detrimental effects on the colonic mucosa, such as oxidative DNA damage, inflammation, and hyperproliferation, significantly contributing to CRC progression in the post-initiation phase (37). Fat consumption has been shown to correlate with increased BAs levels in the intestinal lumen, stimulating the growth and activity of bacteria that convert primary bile acids into secondary ones with strong tumorigenic activity (38). This increase in fecal bile acid excretion is linked to a higher incidence of colorectal cancer (39).

High BAs environments can suppress pancreatic cancer cell proliferation and migration by inducing reactive oxygen species and epithelial-mesenchymal transition, promoting apoptosis. Additionally, elevated plasma BAs levels correlate positively with the risk of colon cancer, while unconjugated and tertiary bile acids do not show this association (40). Despite established links between BAs and colon carcinogenesis, the underlying mechanisms remain unclear.

Early studies by Wilson et al. suggested that Na^+^/K^+^ ATPase might play a role in BAs absorption by ileum cells by maintaining a sodium-electrochemical gradient essential for Na^+^ dependent bile acid transport (12). The apical sodium-dependent bile acid transporter is now known to be primarily responsible for bile acid absorption from the intestinal lumen. Transport occurs electrogenically and Na^+^ dependently with a 2:1 sodium to BAs ratio. This process is driven by the intracellular negative potential and Na^+^/K^+^ ATPase, which maintains the Na^+^ gradient. Substrate binding in the extracellular pocket, along with Na^+^ binding, induces conformational changes in ASBT, opening a cytoplasmic channel for bile acid passage (41).

**Figure 4:**
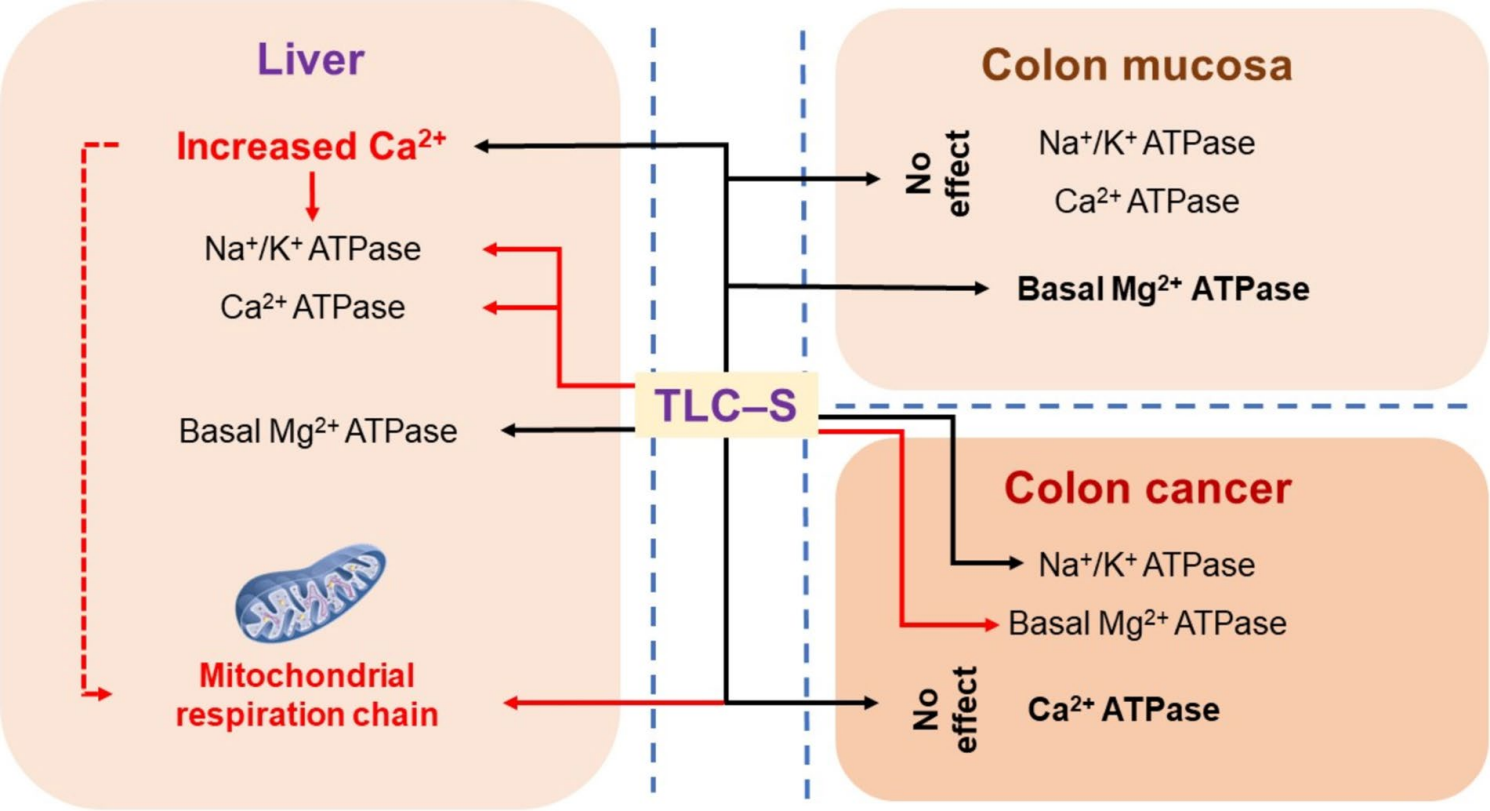
Summary diagram showing the effects of TLC–S in colon cancer, normal colonic mucosa of patients, and the effects in rat liver (red lines mean inhibition, black lines either inhibition or no effect).

We found that TLC–S has different effects on Na^+^/K^+^ ATPase activity in healthy and cancerous colon tissues of patients. In healthy tissue, there was no effect, while in cancerous tissue, TLC–S increased Na^+^/K^+^ ATPase activity. Similarly, TLC–S increased basal Mg^2+^ ATPase activity in normal colonic mucosa samples but inhibited it in cancerous tissue. In the liver, TLC–S inhibited Na^+^/K^+^ ATPase and total Ca^2+^ ATPase activity, but stimulated basal Mg^2+^ ATPase activity. This was accompanied by the release of calcium from intracellular stores and inhibition of mitochondrial respiration rate (using α-ketoglutarate oxidation). We hypothesized that BAs in the colon alter ion transport and affect mucosal ATPase activity. The effect of TLC–S on ATPase activity in colorectal cancer patients’ mucosa was investigated (Figure 4). Adenocarcinoma tissue samples and adjacent healthy mucosa were collected during colonoscopy. We found no significant change in the specific activity of the Na^+^/K^+^ pump in control samples exposed to TLC–S (Figure 4).

Previous studies on rats showed varying effects of different BAs on Na^+^/K^+^ ATPase activity in the large intestine for instance, cholic and taurocholic acids did not inhibit Na^+^/K^+^ ATPase activity at 0.6 mmol/L, unlike glycocholic acid. Taurine derivatives either did not affect or increased Na^+^/K^+^ ATPase activity in the rat ileum (42). Our findings are consistent with these earlier studies.

In colorectal cancer samples, TLC–S incubation resulted in a 1.5-fold increase in Na^+^/K^+^ ATPase activity, suggesting that changes in Na^+^/K^+^ ATPase activity are involved in secondary bile acid carcinogenesis. This effect might be due to taurine in TLC–S, which has been shown to increase Na^+^/K^+^ ATPase activity in the rat brain (43). Bile acids have tissue-specific effects on Na^+^/K^+^ ATPase activity, as seen in rats where bile duct drainage decreased Na^+^/K^+^ ATPase activity in the liver and ileum but not in the jejunum and kidney. Taurocholate administration subsequently increased Na^+^/K^+^ ATPase activity in the liver and ileum but not in the jejunum and kidney (44).

We observed differences in TLC–S effects on Na^+^/K^+^ ATPase activity in healthy and cancerous colonic mucosa (Figure 4). Recent studies indicate that Na^+^/K^+^ ATPase levels are elevated in late-stage colon carcinoma and in cancer cells with an activated Wnt pathway (45). Knockdown of the Na^+^/K^+^ ATPase subunit (ATP1A1) inhibits cell proliferation, migration, and invasion and induces apoptosis in rectal cancer cell lines HT29 and Caco2, suggesting that ATP1A1 regulates tumor progression through the ERK5 signaling pathway (46). Thus, the carcinogenic effect of TLC–S is linked to the increase in Na^+^/K^+^ ATPase activity caused by secondary BAs.

TGR5 is specifically activated by BAs and has conflicting effects on various cancer cells upon activation by TGR5 agonists (47). Lithocholic acid, for instance, inhibits pro-inflammatory cytokine production in the colon via TGR5 activation, reducing rectal cancer development and migration (48). It also alleviates glucose-induced cardiac hypertrophy by enhancing SERCA2a and phosphorylated phospholamban expression in H9C2 cells, thus activating TGR5 (49).

We found that adding TLC–S to colonic mucosa samples increased Ca^2+^ ATPase EPR activity slightly but not significantly. However, in cancerous tissues, Ca^2+^ ATPase EPR activity significantly decreased by 1.4 times with TLC–S addition. This aligns with findings in pancreatic cells where bile acids inhibit Ca^2+^ ATPase, depleting intracellular calcium stores and enhancing inflammation and cell death (50).

Alterations in Ca^2+^ ATPase expression can lead to abnormal cell transformation by affecting Ca^2+^ dependent apoptosis and transcription factors (14). We also studied the effect of TLC–S on PM Ca^2+^ ATPase activity and found no significant changes in control or cancerous samples, though there was a tendency toward decreased activity.

Basal Mg^2+^ ATPase activity, associated with H^+^ translocation in the plasma membrane and endosomal fraction, is a marker of the canalicular membrane in hepatocytes (51, 52). We found a significant increase in basal Mg^2+^ ATPase activity in control colonic mucosa samples under TLC–S influence, consistent with our findings in rat liver subcellular fractions (Figure 4). However, in colorectal cancer cells, TLC–S decreased basal Mg^2+^ ATPase activity by nearly twofold. These contrasting effects of TLC–S on basal Mg^2+^ ATPase activity in healthy and cancerous tissues likely result from cancer cells’ restructuring to avoid apoptosis and activate autophagy.

Na^+^/K^+^ ATPase plays a crucial role in maintaining physiological ion gradients and transmembrane electrical potential in hepatocytes, facilitating sodium-hydrogen exchange and sodium-dependent bile salt absorption (41). TLC–S reduces Na^+^/K^+^ ATPase activity in rat liver subcellular fractions, which correlates with increased intracellular sodium levels observed by other researchers (53). This suggests that TLC–S inhibits BAs secretion by decreasing Na^+^/K^+^ ATPase activity, causing hepatocyte contraction and influencing bile acid transport (54).

TLC–S also decreases Ca^2+^ ATPase activity in rat liver subcellular fractions, likely reflecting a direct effect of calcium depletion (Figure 4). Total Ca^2+^ ATPase activity comprises SERCA and PMCA, with calcium balance maintained by both. Our results align with studies showing that bile acids inhibit SERCA, causing Ca^2+^ release from the endoplasmic reticulum (50). TLC–S likely involves both acidic depots and the EPR, with NAADP-sensitive channels playing a role in Ca^2+^ release. In summary, TLC–S affects rat hepatocytes by releasing calcium from the intracellular store and increasing cytosolic sodium, suppressing Ca^2+^ ATPase and Na^+^/K^+^ ATPase activities while enhancing basal Mg^2+^ ATPase activity. This mechanism involves NAADP sensitive channels, potentially activated by NADPH oxidase, indicating a complex interaction of intracellular stores and ion transporters in response to TLC–S.

We confirmed the previously reported findings by Combettes et al. (26, 27) that TLC–S (50, 100, and 200 μmol/L) induces cytosolic Ca^2+^ signals. Our data further showed that the amplitude of the TLC–S evoked calcium signal is lower compared to the ATP induced signal (Figure 3b), suggesting the involvement of different calcium stores or their interactions. After TLC–S application, hepatocytes retain the ability to respond to ATP, albeit with smaller amplitude and a prolonged plateau phase (Figure 3b). This implies distinct calcium stores are involved in the responses to ATP and TLC–S.

Using the IP_3_R blocker 2-APB (100 μmol/L), we observed that TLC–S induced Ca^2+^ signals were not inhibited by this IP_3_R antagonist (Figure 3c). This indicates that TLC–S mobilizes calcium from a different store, likely an acidic compartment, rather than from the EPR, where IP_3_Rs are located. Additionally, Mamedova et al. (55) demonstrated that deoxycholic and taurocholic acids elevate Ca^2+^ levels in vagus nerve neurons. This calcium increase is dose-dependent and originates from intracellular calcium stores that are insensitive to thapsigargin (55).

We conducted experiments on suspensions of permeabilized hepatocytes pre-loaded with Mag-Fura-2 to monitor the calcium signal within intracellular stores. Our findings revealed that the application of NED-19 (blocker of two–pore channels (TPC), which is NAADP-sensitive Ca^2+^ channels) halved the amount of calcium released by TLC–S. We hypothesize that TLC–S releases calcium from an acidic store containing two-pore channels blocked by NED-19, with the ER also contributing to the release. After TLC–S exposure, thapsigargin was still able to release the stored calcium, indicating the presence of thapsigargin–sensitive calcium in the EPR.

We propose that acidic calcium stores play a crucial role in replenishing the EPR with calcium. The observed slowing of calcium release from the EPR after NED-19 pretreatment, induced by TLC–S and thapsigargin, is likely due to the blockade of TPC at contact sites between NAADP sensitive acidic stores and the EPR.

TLC–S has been shown to cause hyperosmolarity in hepatocytes due to endosomal acidification and activation of NADPH oxidase isoforms. Extracellular messengers like angiotensin II can activate NADPH oxidase, leading to an increase in NAADP from NADPH. NADPH oxidase can increase NADPH levels, which are then converted to NAADP, a nucleotide that releases calcium from the endolysosomal system or acidic stores (56). Given that NED-19 (an NAADP antagonist) reduces the fraction of Ca^2+^ released by TLC–S, we directly confirmed the involvement of acidic stores in the TLC–S effect.

In conclusion, NAADP-sensitive acidic Ca^2+^ stores are involved in TLC–S induced cytosolic Ca^2+^ signals in rodent liver cells, leading to decreased calcium levels in the EPR of mouse hepatocytes. Therefore, the mechanism of TLC–S induced Ca^2+^ release is mediated by NAADP-sensitive Ca^2+^ channels, which are blocked by NED-19. It is proposed that TLC–S stimulates NAADP production.

We found in studying the impact of TLC–S on rat liver mitochondria respiration parameters, a significant decrease in V_3_ respiration rate during α-ketoglutarate oxidation was observed. As it is an NAD dependent substrate, it undergoes oxidation under normal mitochondrial activity. This indicates that TLC–S affects mitochondrial function, with a notable decrease in V_3_ state indicators signifying reduced operational speed and power.

The obtained data indicate that TLC–S does not cause significant changes in respiratory control ratios (V_3_/V_2_, V_3_/V_4_) or oxidative phosphorylation parameters (ADP/O, Vf, Tf) during the oxidation of NAD-dependent (α-ketoglutarate) and FAD-dependent (succinate) substrates. Although slight reductions in respiratory control and increases in ADP/O were observed, these changes were not statistically significant. TLC–S affects oxidative phosphorylation efficiency and speed differently depending on the substrate. While there is a trend to increase in ADP/O ratio for both α-ketoglutarate and succinate, indicating a tendency to improving ATP synthesis efficiency, the changes in phosphorylation rate (Vf) and phosphorylation time (Tf) are inconsistent.

The observed inhibition of Na^+^/K^+^ ATPase and total Ca^2+^ ATPase activity by TLC–S leads to an increase in cytosolic calcium, which may affect mitochondrial respiration. We observed a slowdown in the respiration rate of isolated mitochondria in state V_3_ in rat liver under the influence of TLC–S during α-ketoglutarate oxidation.

Our findings are consistent with those of Voronina et al. (57), who demonstrated that TLC–S induces significant mitochondrial depolarization in pancreatic acinar cells. This depolarization is likely driven by an increase in cytosolic calcium. These results suggest that TLC–S may contribute to mitochondrial dysfunction, potentially playing a role in the development of acute pancreatitis (57).

TLC–S affects mitochondrial membrane potential and calcium levels in cells, which mediates its impact on reactive oxygen species (ROS). In particular, it has been shown that TLC–S causes a significant increase in cytosolic calcium levels in pancreatic cells, which depends on external sodium and calcium influx (58). This calcium overload in mitochondria is accompanied by a decrease in mitochondrial membrane potential and, as a result, increased ROS production in mitochondria (59). In this study, it was found that TLC–S induces ROS formation in mitochondria through a calcium-dependent mechanism, as pre-blocking calcium signals with BAPTA–AM reduced ROS production. The connection between calcium elevation and ROS induction by TLC–S was also demonstrated in liver cells (60).

Summarizing the mechanisms by which TLC–S effects in rodent hepatocytes (Figure 4), we observe calcium release from intracellular stores and increased cytosolic sodium, consistent with findings from other studies. The TLC–S induced cytosolic Ca^2+^ signals in mouse hepatocytes were attributed to calcium release from acidic stores. The released calcium is suppressing the activities of Ca^2+^ ATPase and Na^+^/K^+^ ATPase, while increasing the basal activity of Mg^2+^ ATPase in rat liver subcellular fractions at the same time this leads to decreasing of respiration rate of mitochondria.

In patient colon cancer tissue, TLC–S exhibits an opposite effect on ATPase activity compared to rat liver, which may be linked to the mechanisms of cancer development. TLC–S increased Na^+^/K^+^ ATPase activity in colorectal cancer samples from patients, while reducing basal Mg^2+^ ATPase activity in normal colon mucosa tissue from the same patients.

## Conclusion

The study on the TLC–S on ATPase activity in colorectal cancer tissues and rodent liver revealed significant alterations in different ATPase types, which may be crucial for understanding the mechanisms of carcinogenesis. TLC–S induced an increase in Na^+^/K^+^ ATPase activity in colorectal cancer tissues, suggesting a potential role of this enzyme in maintaining ionic homeostasis and possibly contributing to cancer progression. The absence of a similar effect in healthy colon tissues confirms the tissue-specific action of TLC–S and indicates that changes in Na^+^/K^+^ ATPase activity may be specific to malignant cells. The study also demonstrated that TLC–S significantly decreases basal Mg^2+^ ATPase activity in cancerous tissues, while in healthy colon tissues the activity of this enzyme is increased. This may indicate adaptive changes in cancer cells aimed at avoiding apoptosis and maintaining their viability under stress conditions.

In rodent liver, TLC–S reduced Ca^2+^ ATPase and Na^+^/K^+^ ATPase activity, leading to an increase in cytosolic calcium concentration and altered mitochondrial function. The observed decrease in mitochondrial respiration rate under the influence of TLC–S suggests an impact on the metabolic activity of liver cells. Additionally, TLC–S caused the release of calcium from intracellular stores in mouse hepatocytes, which was partially inhibited by the NAADP antagonist, NED-19. This indicates that TLC–S modulates the activity of calcium channels and ion transporters, affecting calcium exchange and intracellular signaling pathways.

Thus, TLC–S exhibits divergent effects on ATPase activity in healthy and cancerous colon tissues, which may be linked to cancer development mechanisms. In rodent liver, TLC–S affects mitochondrial activity and ATPase function, leading to altered ionic homeostasis, which may be significant for understanding the role of bile acids in liver pathology and colorectal cancer. These findings could contribute to further research into the mechanisms by which bile acids influence cancer development and other pathological conditions, potentially leading to therapeutic applications.

## Data Availability

The raw data supporting the conclusions of this article will be made available by the authors, without undue reservation.

## References

[1] Gillard J Leclercq IA. Biological tuners to reshape the bile acid pool for therapeutic purposes in non-alcoholic fatty liver disease. Clin Sci. (2023) 137:65–85. doi: 10.1042/CS20220697, PMID: 36601783 PMC9816373

[2] Ticho AL Malhotra P Dudeja PK Gill RK Alrefai WA. Intestinal absorption of bile acids in health and disease. Comprehen. Physiol. (2019) 10:21–56. doi: 10.1002/cphy.c190007, PMID: 31853951 PMC7171925

[3] Tveter KM Mezhibovsky E Wu Y Roopchand DE. Bile acid metabolism and signaling: emerging pharmacological targets of dietary polyphenols. Pharmacol Ther. (2023) 248:108457. doi: 10.1016/j.pharmthera.2023.108457, PMID: 37268113 PMC10528343

[4] Bernstein C Holubec H Bhattacharyya AK Nguyen H Payne CM Zaitlin B. Carcinogenicity of deoxycholate, a secondary bile acid. Arch Toxicol. (2011) 85:863–71. doi: 10.1007/s00204-011-0648-7, PMID: 21267546 PMC3149672

[5] Ridlon JM Harris SC Bhowmik S Kang D-J Hylemon PB. Consequences of bile salt biotransformations by intestinal bacteria. Gut Microbes. (2016) 7:22–39. doi: 10.1080/19490976.2015.1127483, PMID: 26939849 PMC4856454

[6] Bernstein H Bernstein C Payne CM Dvorakova K Garewal H. Bile acids as carcinogens in human gastrointestinal cancers. Mutat Res. (2005) 589:47–65. doi: 10.1016/j.mrrev.2004.08.001 15652226

[7] Li J Chen D Shen M. Tumor microenvironment shapes colorectal Cancer progression, metastasis, and treatment responses. Front Med. (2022) 9:869010. doi: 10.3389/fmed.2022.869010, PMID: 35402443 PMC8984105

[8] He Q Wu J Ke J Zhang Q Zeng W Luo Z. Therapeutic role of ursodeoxycholic acid in colitis-associated cancer via gut microbiota modulation. Mol Ther. (2023) 31:585–98. doi: 10.1016/j.ymthe.2022.10.014, PMID: 38556635 PMC9931610

[9] Jia W Xie G Jia W. Bile acid–microbiota cross-talk in gastrointestinal inflammation and carcinogenesis. Nat Rev Gastroenterol Hepatol. (2018) 15:111–28. doi: 10.1038/nrgastro.2017.119, PMID: 29018272 PMC5899973

[10] Da Silva CI Gonçalves-de-Albuquerque CF De Moraes BPT Garcia DG Burth P. Na/K-ATPase: their role in cell adhesion and migration in cancer. Biochimie. (2021) 185:1–8. doi: 10.1016/j.biochi.2021.03.002, PMID: 33713729

[11] Song Y Lee S-Y Kim S Choi I Kim S-H Shum D. Inhibitors of Na+/K+ ATPase exhibit antitumor effects on multicellular tumor spheroids of hepatocellular carcinoma. Sci Rep. (2020) 10:5318. doi: 10.1038/s41598-020-62134-4, PMID: 32210281 PMC7093469

[12] Wilson FA Treanor LL. Studies of relationship among bile-acid uptake, Na+, K+-ATPase, and Na+ gradient in isolated cells from rat ileum. Gastroenterology. (1981) 81:54–60. doi: 10.1016/0016-5085(81)90652-1, PMID: 6165641

[13] Chen D Song M Mohamad O Yu SP. Inhibition of Na+/K+-ATPase induces hybrid cell death and enhanced sensitivity to chemotherapy in human glioblastoma cells. BMC Cancer. (2014) 14:716. doi: 10.1186/1471-2407-14-716, PMID: 25255962 PMC4190379

[14] Aung CS Ye W Plowman G Peters AA Monteith GR Roberts-Thomson SJ. Plasma membrane calcium ATPase 4 and the remodeling of calcium homeostasis in human colon cancer cells. Carcinogenesis. (2009) 30:1962–9. doi: 10.1093/carcin/bgp223, PMID: 19755660

[15] Chen J Sitsel A Benoy V Sepúlveda MR Vangheluwe P. Primary active ca ^2+^ transport Systems in Health and Disease. Cold Spring Harb Perspect Biol. (2020) 12:a035113. doi: 10.1101/cshperspect.a035113, PMID: 31501194 PMC6996454

[16] Peters AA Milevskiy MJG Lee WC Curry MC Smart CE Saunus JM. The calcium pump plasma membrane Ca2+-ATPase 2 (PMCA2) regulates breast cancer cell proliferation and sensitivity to doxorubicin. Sci Rep. (2016) 6:25505. doi: 10.1038/srep25505, PMID: 27148852 PMC4857793

[17] Chemaly ER Troncone L Lebeche D. SERCA control of cell death and survival. Cell Calcium. (2018) 69:46–61. doi: 10.1016/j.ceca.2017.07.001, PMID: 28747251 PMC5748262

[18] Bychkova S Bychkov M Dordevic D Vítězová M Rittmann SK-MR Kushkevych I. Bafilomycin A1 molecular effect on ATPase activity of subcellular fraction of human colorectal Cancer and rat liver. IJMS. (2024) 25:1657. doi: 10.3390/ijms25031657, PMID: 38338935 PMC10855383

[19] Bychkova SV Stasyshyn AR Bychkov MA. The role of bafilomycin as a therapeutic agent in the modulation of endo-lysosomal store of rat hepatocytes. Med perspekt. (2022) 27:22–6. doi: 10.26641/2307-0404.2022.3.265768

[20] Kushkevych I Bychkov M Bychkova S Gajdács M Merza R Vítězová M. ATPase activity of the subcellular fractions of colorectal Cancer samples under the action of nicotinic acid adenine dinucleotide phosphate. Biomedicines. (2021) 9:1805. doi: 10.3390/biomedicines9121805, PMID: 34944620 PMC8698369

[21] Kushkevych I Fafula R Parák T Bartoš M. Activity of Na+/K+-activated Mg2+−dependent ATP-hydrolase in the cell-free extracts of the sulfate-reducing bacteria *Desulfovibrio piger* Vib-7 and Desulfomicrobium sp. Rod-9. Acta Vet Brno. (2015) 84:3–12. doi: 10.2754/avb201585010003

[22] Hreniukh V Bychkova S Kulachkovsky O Babsky A. Effect of bafilomycin and NAADP on membrane-associated ATPases and respiration of isolated mitochondria of the murine Nemeth-Kellner lymphoma: mitochondria and ATPase activities in lymphoma. Cell Biochem Funct. (2016) 34:579–87. doi: 10.1002/cbf.3231, PMID: 27862060

[23] Babsky A Doliba N Doliba N Savchenko A Wehrli S Osbakken M. Na ^+^ effects on mitochondrial respiration and oxidative phosphorylation in diabetic hearts. Exp Biol Med (Maywood). (2001) 226:543–51. doi: 10.1177/153537020122600606, PMID: 11395924

[24] Chance B Williams GR. Respiratory enzymes in oxidative phosphorylation. I. Kinetics of oxygen utilization. J Biol Chem. (1955) 217:383–93. doi: 10.1016/S0021-9258(19)57189-7 13271402

[25] Nicholls D. G. (2013). Bioenergetics. Available online at: https://shop.elsevier.com/books/bioenergetics/nicholls/978-0-12-388425-1 (Accessed July 24, 2024)

[26] Combettes L Berthon B Doucet E Erlinger S Claret M. Characteristics of bile acid-mediated Ca2+ release from permeabilized liver cells and liver microsomes. J Biol Chem. (1989) 264:157–67. doi: 10.1016/S0021-9258(17)31237-1, PMID: 2783315

[27] Combettes L Dumont M Berthon B Erlinger S Claret M. Release of calcium from the endoplasmic reticulum by bile acids in rat liver cells. J Biol Chem. (1988) 263:2299–303. doi: 10.1016/S0021-9258(18)69205-1, PMID: 3257491

[28] Dordevic D Capikova J Dordevic S Tremlová B Gajdács M Kushkevych I. Sulfur content in foods and beverages and its role in human and animal metabolism: a scoping review of recent studies. Heliyon. (2023) 9:e15452. doi: 10.1016/j.heliyon.2023.e15452, PMID: 37123936 PMC10130226

[29] Dordević D Jančíková S Vítězová M Kushkevych I. Hydrogen sulfide toxicity in the gut environment: Meta-analysis of sulfate-reducing and lactic acid bacteria in inflammatory processes. J Adv Res. (2020) 27:55–69. doi: 10.1016/j.jare.2020.03.003, PMID: 33318866 PMC7728594

[30] Kushkevych I Cejnar J Treml J Dordević D Kollar P Vítězová M. Recent advances in metabolic pathways of sulfate reduction in intestinal Bacteria. Cells. (2020) 9:698. doi: 10.3390/cells9030698, PMID: 32178484 PMC7140700

[31] Kushkevych I Dordević D Kollár P. Analysis of physiological parameters of Desulfovibrio strains from individuals with colitis. Open Life Sci. (2019) 13:481–8. doi: 10.1515/biol-2018-0057, PMID: 33817117 PMC7874683

[32] Kushkevych I Dordević D Vítězová M. Possible synergy effect of hydrogen sulfide and acetate produced by sulfate-reducing bacteria on inflammatory bowel disease development. J Adv Res. (2020) 27:71–8. doi: 10.1016/j.jare.2020.03.007, PMID: 33318867 PMC7728581

[33] Kushkevych I Dordević D Vítězová M Kollár P. Cross-correlation analysis of the Desulfovibrio growth parameters of intestinal species isolated from people with colitis. Biologia. (2018) 73:1137–43. doi: 10.2478/s11756-018-0118-2

[34] Kushkevych I Leščanová O Dordević D Jančíková S Hošek J Vítězová M. The sulfate-reducing microbial communities and Meta-analysis of their occurrence during diseases of small-large intestine Axis. JCM. (2019) 8:1656. doi: 10.3390/jcm8101656, PMID: 31614543 PMC6832292

[35] Kushkevych I Martínková K Mráková L Giudici F Baldi S Novak D. Comparison of microbial communities and the profile of sulfate-reducing bacteria in patients with ulcerative colitis and their association with bowel diseases: a pilot study. Microb Cell. (2024) 11:79–89. doi: 10.15698/mic2024.03.817, PMID: 38486888 PMC10939707

[36] Kushkevych I Vítězová M Fedrová P Vochyanová Z Paráková L Hošek J. Kinetic properties of growth of intestinal sulphate-reducing bacteria isolated from healthy mice and mice with ulcerative colitis. Acta Vet Brno. (2017) 86:405–11. doi: 10.2754/avb201786040405

[37] Gadaleta RM Garcia-Irigoyen O Moschetta A. Bile acids and colon cancer: is FXR the solution of the conundrum? Mol Asp Med. (2017) 56:66–74. doi: 10.1016/j.mam.2017.04.002, PMID: 28400119

[38] Ocvirk S O’Keefe SJ. Influence of bile acids on colorectal Cancer risk: potential mechanisms mediated by diet-gut microbiota interactions. Curr Nutr Rep. (2017) 6:315–22. doi: 10.1007/s13668-017-0219-5, PMID: 29430336 PMC5802424

[39] Stamp DH. Three hypotheses linking bile to carcinogenesis in the gastrointestinal tract: certain bile salts have properties that may be used to complement chemotherapy. Med Hypotheses. (2002) 59:398–405. doi: 10.1016/S0306-9877(02)00125-1, PMID: 12208178

[40] Kühn T Stepien M López-Nogueroles M Damms-Machado A Sookthai D Johnson T. Prediagnostic plasma bile acid levels and Colon Cancer risk: a prospective study. JNCI J Natl Cancer Inst. (2020) 112:516–24. doi: 10.1093/jnci/djz166, PMID: 31435679 PMC7225675

[41] Durník R Šindlerová L Babica P Jurček O. Bile acids transporters of enterohepatic circulation for targeted drug delivery. Molecules. (2022) 27:2961. doi: 10.3390/molecules27092961, PMID: 35566302 PMC9103499

[42] Hafkenscheid JCM. Influence of bile acids on the (Na+−K+)-activated- and Mg2+−activated ATPase of rat colon. Pflugers Arch. (1977) 369:203–6. doi: 10.1007/BF00582185, PMID: 142961

[43] El Idrissi A. (2019). “Taurine regulation of neuroendocrine function,” in Taurine 11, eds. Hu J. Piao F. Schaffer S. W. Idrissi A. El Wu J.-Y. (Singapore: Springer Singapore), 977–985. 10.1007/978-981-13-8023-5_81 31468461

[44] Simon FR Sutherland E Sutherland J. Selective modulation of hepatic and ileal Na+-K+-ATPase by bile salts in the rat. Am J Physiol Gastrointest Liver Physiol. (1988) 254:G761–7. doi: 10.1152/ajpgi.1988.254.5.G761 2834964

[45] Tejeda-Muñoz N Azbazdar Y Sosa EA Monka J Wei P-S Binder G. Na,K-ATPase activity promotes macropinocytosis in colon cancer via Wnt signaling. Biol Open. (2024) 13:bio060269. doi: 10.1242/bio.060269, PMID: 38713004 PMC11139033

[46] Sumiyoshi S Shiozaki A Kosuga T Simizu H Kudo M Kiuchi J. Functional analysis and clinical importance of ATP1A1 in Colon Cancer. Ann Surg Oncol. (2023) 30:6898–910. doi: 10.1245/s10434-023-13779-8, PMID: 37407874

[47] Qi Y Duan G Wei D Zhao C Ma Y. The bile acid membrane receptor TGR5 in Cancer: friend or foe? Molecules. (2022) 27:5292. doi: 10.3390/molecules27165292, PMID: 36014536 PMC9416356

[48] Zhang H Xu H Zhang C Tang Q Bi F. Ursodeoxycholic acid suppresses the malignant progression of colorectal cancer through TGR5-YAP axis. Cell Death Discov. (2021) 7:207. doi: 10.1038/s41420-021-00589-8, PMID: 34365464 PMC8349355

[49] Cheng K-C Chang W-T Kuo FY Chen Z-C Li Y Cheng J-T. TGR5 activation ameliorates hyperglycemia-induced cardiac hypertrophy in H9c2 cells. Sci Rep. (2019) 9:3633. doi: 10.1038/s41598-019-40002-0, PMID: 30842472 PMC6403401

[50] Kim JY Kim KH Lee JA Namkung W Sun A Ananthanarayanan M. Transporter-mediated bile acid uptake causes Ca2+−dependent cell death in rat pancreatic acinar cells. Gastroenterology. (2002) 122:1941–53. doi: 10.1053/gast.2002.33617, PMID: 12055600

[51] Kosterin SO Veklich TO Pryluts’kyi Iu I. Kinetic interpretation of the original pH-dependence of enzymatic activity of „basal” Mg2+ ATPase of the smooth muscle sarcolemma. Ukr Biokhim Zh. (2005) 2005:37–45. 19618740

[52] Luu-The V Goffeau A Thinès-Sempoux D. Rat liver plasma membrane Ca2+− or Mg2+−activated ATPase. Evidence for proton movement in reconstituted vesicles. Biochimica et Biophysica Acta. (1987) 904:251–8. doi: 10.1016/0005-2736(87)90374-9 2822118

[53] Voronina SG Gryshchenko OV Gerasimenko OV Green AK Petersen OH Tepikin AV. Bile acids induce a cationic current, depolarizing pancreatic acinar cells and increasing the intracellular Na+ concentration. J Biol Chem. (2005) 280:1764–70. doi: 10.1074/jbc.M410230200, PMID: 15536077

[54] Becker S Reinehr R Graf D Vom Dahl S Häussinger D. Hydrophobic bile salts induce hepatocyte shrinkage via NADPH oxidase activation. Cell Physiol Biochem. (2007) 19:89–98. doi: 10.1159/000099197, PMID: 17310103

[55] Mamedova E Árting LB Rekling JC. Bile acids induce Ca2+ signaling and membrane permeabilizations in vagal nodose ganglion neurons. Biochem Biophys Rep. (2022) 31:101288. doi: 10.1016/j.bbrep.2022.101288, PMID: 35669985 PMC9162955

[56] Billington RA Thuring JW Conway SJ Packman L Holmes AB Genazzani AA. Production and characterization of reduced NAADP (nicotinic acid-adenine dinucleotide phosphate). Biochem J. (2004) 378:275–80. doi: 10.1042/bj20031284, PMID: 14606955 PMC1223936

[57] Voronina SG Barrow SL Gerasimenko OV Petersen OH Tepikin AV. Effects of Secretagogues and bile acids on mitochondrial membrane potential of pancreatic acinar cells. J Biol Chem. (2004) 279:27327–38. doi: 10.1074/jbc.M311698200, PMID: 15084611

[58] Ferdek PE Jakubowska MA Gerasimenko JV Gerasimenko OV Petersen OH. Bile acids induce necrosis in pancreatic stellate cells dependent on calcium entry and sodium-driven bile uptake. J Physiol. (2016) 594:6147–64. doi: 10.1113/JP272774, PMID: 27406326 PMC5088250

[59] Booth DM Mukherjee R Sutton R Criddle DN. Calcium and reactive oxygen species in acute pancreatitis: friend or foe? Antioxid Redox Signal. (2011) 15:2683–98. doi: 10.1089/ars.2011.3983, PMID: 21861696 PMC3183657

[60] Karimian G Buist-Homan M Mikus B Henning RH Faber KN Moshage H. Angiotensin II protects primary rat hepatocytes against bile salt-induced apoptosis. PLoS One. (2012) 7:e52647. doi: 10.1371/journal.pone.0052647, PMID: 23300732 PMC3530435

